# Unlocking species identity: geometric morphometrics of head and thorax shapes in invasive and non-invasive quarantine-significant thrips (Thysanoptera: Terebrantia)

**DOI:** 10.3389/finsc.2025.1558242

**Published:** 2025-03-06

**Authors:** Allan H. Smith-Pardo, Laura M. Pérez, Hugo A. Benítez

**Affiliations:** ^1^ United States Department of Agriculture (USDA)-Animal and Plant Health Inspection Service (APHIS)-Plant Protection and Quarantine (PPQ)-Science and Technology (S&T), Pest Identification Technology Laboratory (PITL), Sacramento, CA, United States; ^2^ Departamento de Ingeniería Industrial y de Sistemas, Universidad de Tarapacá, Arica, Chile; ^3^ Laboratorio de Ecología y Morfometría Evolutiva, Centro de Investigación de Estudios Avanzados del Maule, Universidad Católica del Maule, Talca, Chile; ^4^ Research Ring in Pest Insects and Climate Change (PIC2), Santiago, Chile; ^5^ Millennium Institute Biodiversity of Antarctic and Sub-Antarctic Ecosystems (BASE), Santiago, Chile; ^6^ Cape Horn International Center (CHIC), Centro Universitario Cabo de Hornos, Universidad de Magallanes, Puerto Williams, Chile

**Keywords:** quarantine significant, pests, taxonomy, morphometrics, shape variation, phenotype

## Abstract

This study use landmark based geometric morphometrics (GM) of the head and the thorax on eight species of thrips of the species-rich genus *Thrips*. Among the selected species, four were classified as common and not significant, while four were identified as quarantine-significant and agriculturally important in the USA. The results indicate the potential for using both sets of landmarks, which, in some cases, were complementary. When one set did not reveal significant differences in shape, the other provided valuable insights. The geometric morphometric analysis of the selected landmarks revealed statistically significant differences in head morphology and the configuration of setal insertion points on the mesothorax and metathorax. Principal component analysis (PCA) served as the primary method to examine the ordinal distribution of the eight species within the morphospace. The analysis highlighted *T. australis* and *T. angusticeps* as the most morphologically distinct species in terms of head shape, while *T. nigropilosus*, *T. obscuratus*, and *T. hawaiiensis* exhibited the greatest divergence in thoracic morphology. The results further demonstrate the potential of geometric morphometric (GM) methods for identifying taxa that are challenging to distinguish using traditional taxonomy based on external morphology. This is particularly relevant for morphologically conservative taxa, such as thrips with minimal or no wing venation (a feature often used in GM studies of winged insects), species complexes (e.g., *T. hawaiiensis* and related species examined in this study), and taxa exhibiting morphological similarity due to convergent evolution associated with shared ecological niches.

## 1 Introduction

Thysanoptera (commonly named thrips) comprises more than 7000 described species. Currently, nearly 850 genera are accepted (including 65 genera of fossils), the suprageneric classification remains in flux, and the evolutionary relationships among groups are still obscure, resulting in nearly half of the recognized genera being monotypic/monobasic (there are almost 440 genera with only one species). In addition, around 230 genera include no more than five species, and only ten genera include more than 100 species, in which the genus *Thrips* is included (1, 2).

The suborder Terebrantia comprises eight extant families, the largest of which is Thripidae, which includes more than 2000 species in nearly 290 genera (1). The family Thripidae has been generally divided into four subfamilies (Dendrothripinae, Sericothripinae, Panchaetothripinae, and Thripinae), of which Thripinae is the largest, with more than 1800 species and over 200 genera (3). According to Masumoto and Okajima (4), even though the suprageneric relationships within the subfamily Thripinae are unclear, some monophyletic groups are recognized (5–7) one of which is the genus *Thrips*, which are among the most important agricultural pests globally because of the damage inflicted by their oviposition, feeding, and their ability to transmit plant viruses (8).

With over 280 species of *Thrips* worldwide, many of which are common pests and vectors of viruses, the need for accurate identification is not just critical but crucial. However, their identification can be challenging due to the lack of comprehensive information about the biology, distribution, and variation within and between species. This difficulty in identification can have severe consequences in the regular trade or agricultural commodities, particularly in the USA, where the most intercepted group of thrips belong to this genus (9). Thrips species have successfully colonized a wide range of natural and non-natural habitats, demonstrating remarkable ecological adaptability. Their presence ranges from forests and grasslands to agricultural and urban landscapes, where they occupy diverse niches including foliage, flowers, bark, and leaf litter (10). However, collecting specimens for identification poses significant challenges due to their small size, rapid dispersal, and tendency to occupy hidden microhabitats. These difficulties highlight the need for targeted sampling techniques, such as suction traps, sticky cards, and Berlese-Tullgren funnels, which vary in efficiency depending on species habitat and behavior (10). Different methodologies have been used to identify Thrips species morphologically, focusing on analyzing phenotypic traits to distinguish complexes of cryptic species (11). A comparison of traditional morphological methods and modern approaches including geometric morphometrics, molecular techniques, and biochemical analyses has enhanced the recognition of *Thrips* species (11, 12). This comparison highlights the strengths and limitations of each method, emphasizing how geometric morphometrics complements traditional techniques by quantifying subtle morphological differences that are difficult to discern visually. Musa et al. (13) use traditional morphometrics to differentiate between subspecies of *Thrips tabaci* based on various body traits. GM methods have been used across multiple insect taxa, including beetles. For example, Cáceres et al. (14) applied these techniques alongside traditional taxonomic methods to redefine the generic limits of Syndesini beetles by analyzing key morphological traits, such as mandibles and pronotal tubercles. Their study provided statistical support for distinguishing genera and clarified biogeographic patterns associated with Gondwanan vicariance. Additionally, GM tools have been employed to identify developmental instability by examining organismal symmetry, which can reflect the effects of various types of stress (15).

In this study, we explore the potential of geometric morphometrics as a complementary tool for species identification within the genus *Thrips*. By integrating this approach with traditional taxonomy based on external morphology, we aim to facilitate a more rapid and cost-effective method for distinguishing species of quarantine significance from those frequently intercepted but of lesser concern.

## 2 Materials and methods

For this study, eight commonly intercepted species of the genus *Thrips* at U.S. ports of entry were selected. Half of these species are considered of quarantine significance (not present, or with limited distribution and under eradication), while the other half are classified as not quarantine-significant (already present in the continental USA). All specimens analyzed were slide-mounted adult females with high-resolution images. These images were obtained from USDA-APHIS-PPQ, where USDA specialists previously identified the specimens and included them in the ImageID database. Additionally, other specialists in the group verified that the few images sourced from other websites were correctly identified, with USDA specialists reviewing and confirming their accuracy (**Table 1**).

**Table 1:** Morphometric Procrustes and Mahalanobis distances of head shape between *Thrips* species, with respective permutation comparison p-values.

Distances	Procrustes distances						
Species	*Thrips angusticeps*	*Thrips australis*	*Thrips flavus*	*Thrips hawaiiensis*	*Thrips nigropilosus*	*Thrips obscuratus*	*Thrips palmi*
*Thrips australis*	0.1207						
*Thrips flavus*	0.0827	0.1068					
*Thrips hawaiiensis*	0.0834	0.0671	0.074				
*Thrips nigropilosus*	0.1083	0.0786	0.0992	0.0669			
*Thrips obscuratus*	0.0999	0.0837	0.0886	0.0847	0.0748		
*Thrips palmi*	0.0918	0.0777	0.0762	**0.0331**	0.0764	0.1016	
*Thrips setosus*	0.0629	0.098	0.0743	0.0867	0.0827	0.0749	0.0942
	Mahalanobis distances						
*Thrips australis*	13.300						
*Thrips flavus*	9.455	9.591					
*Thrips hawaiiensis*	8.981	5.686	7.196				
*Thrips nigropilosus*	11.420	9.566	9.485	5.9922			
*Thrips obscuratus*	8.002	8.170	8.198	4.247	7.010		
*Thrips palmi*	9.203	6.233	7.129	2.900	6.581	4.401	
*Thrips setosus*	7.772	8.337	5.066	5.135	7.020	5.678	5.675

The bold number represents the non-significative value of the permutation test.

**Figure 1:**
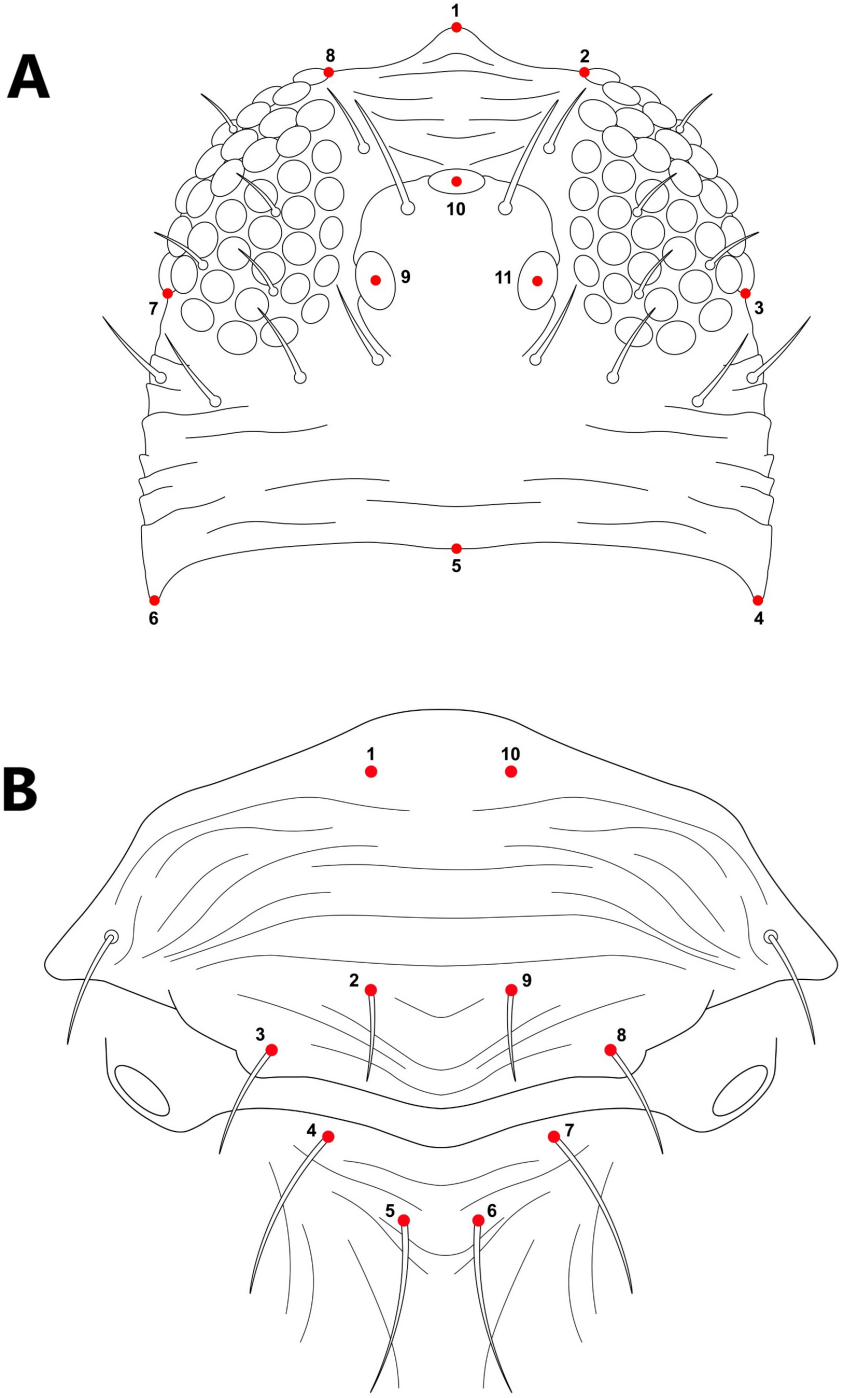
Landmark representation of **(A)** head morphology with 11 landmarks and **(B)** the thorax with 10 landmarks around their setae in the mesonotum and metanotum.

Fifty-eight and fifty specimens were used for the head and thorax, respectively; images were processed using Photoshop vs 26.0 (2025, Adobe Creative Cloud), cropped to the target tagma, and enhanced through higher contrast and sharpening. Landmarks placing were processed using the software TPS Dig2 v2.17 (16). For the head, 11 landmarks were digitized as illustrated in **Figure 1A**, and for the thorax, we used the distribution of 10 setae in the meso and metanotum (**Figure 1B**). The cartesian coordinates from the landmarks were processed using a Procrustes fit analysis in the software MorphoJ 1.07a which standardized the samples by removing the effects of size, position, and rotation (17). Head and thorax shape variation were analyzed using principal component analysis (PCA) based on the covariance matrix of individual shapes. An average shape covariance matrix was computed to identify species-specific shape characteristics, followed by its corresponding PCA (18, 19). These analyses were employed to provide a clear visualization of the morphospace. Differences between groups were evaluated using a permutation test with 10,000 iterations, incorporating Mahalanobis and Procrustes distances. Procrustes distances measure the absolute magnitude of shape deviations from the centroid size, while Mahalanobis distances account for variance and indicate how distinct an individual is relative to others in the sample (20, 21). Together, these metrics summarize overall patterns of similarity and highlight shape differences among species (22, 23). All the morphometrics analyses were performed using the package geomorph (24) and ggplot2 (25) in software R and MorphoJ 1.07a (26).

## 3 Results

### 3.1 Head shape

**Figure 2:**
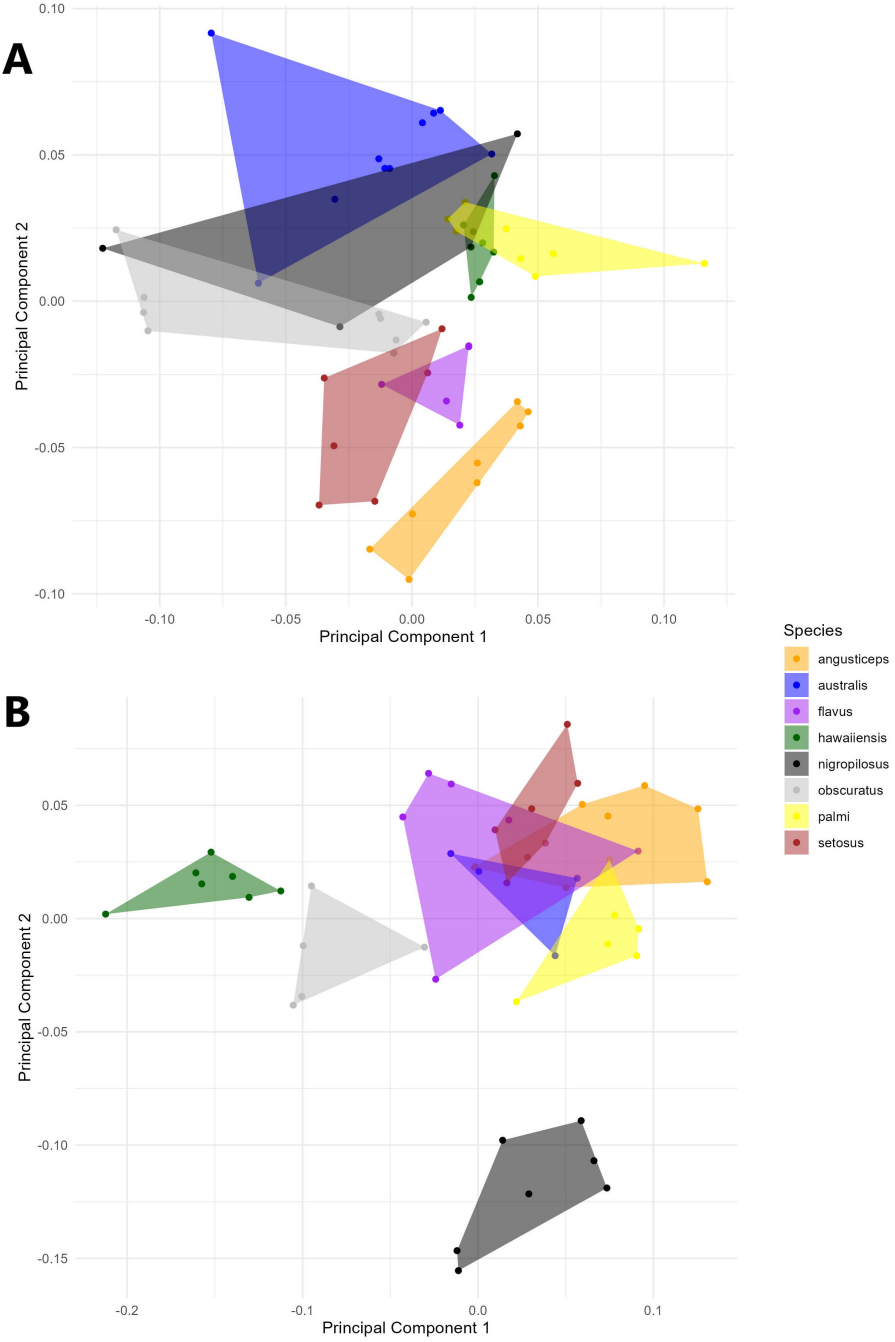
Principal component analysis of the *Thrips* species every color represent a single specie for **(A)** the head and **(B)** the thorax.

**Figure 3:**
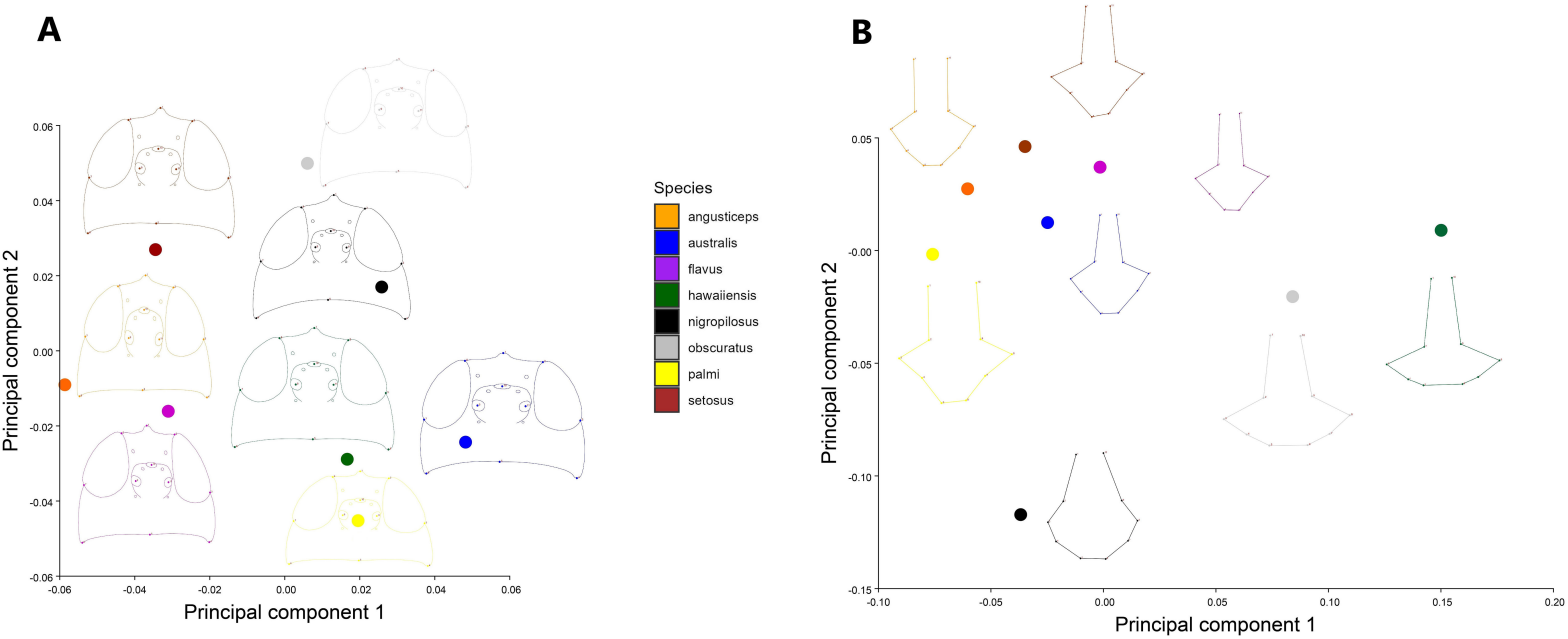
Principal component analysis of the average shape for **(A)** the head and **(B)** the thorax. The points on the morphospace represent the average shape for each species of *Thrips*.

A PCA of the covariance matrix showed that a particular head shape is directly related to each species. The first three PCs accounted for over 73% (PC1 = 33.07%; PC2 = 25.94%; PC3 = 14.02%) of the total head shape variation. The PCA reveals a clustering of individuals, primarily driven by variance along PC2. The extremes of the vertical axis are notably characterized by specimens of *T. angusticeps* and *T. australis*. In the central region of the morphospace, overlapping groups were observed, including *T. hawaiiensis* and *T. palmi*, as well as *T. nigropilosus* and *T. obscuratus* (**Figure 2A**). The PCA illustrates the average head shape of each species, showing clear differentiation at the two extremes of the PCA, with *T. australis* and *T. angusticeps*. These species exhibit a flattened head shape characterized by opposing vectorial movements of landmarks #1 and #5 (head height) and #4 and #8 (head width). Similar shapes were observed among *T. flavus*, *T. setosus*, and *T. angusticeps*, located in the mid-left of the morphospace. Meanwhile, *T. palmi*, *T. australis*, and *T. hawaiiensis* occupied the lower-right extreme, generally displaying elongated, semi-oval shapes (**Figure 3A**). ANOVA analyses revealed no significant differences in size across the species (centroid size: F = 0.99, p = 0.4480), but significant differences in shape (Procrustes distances: F = 7.89, p < 0.0001).

**Figure 4:**
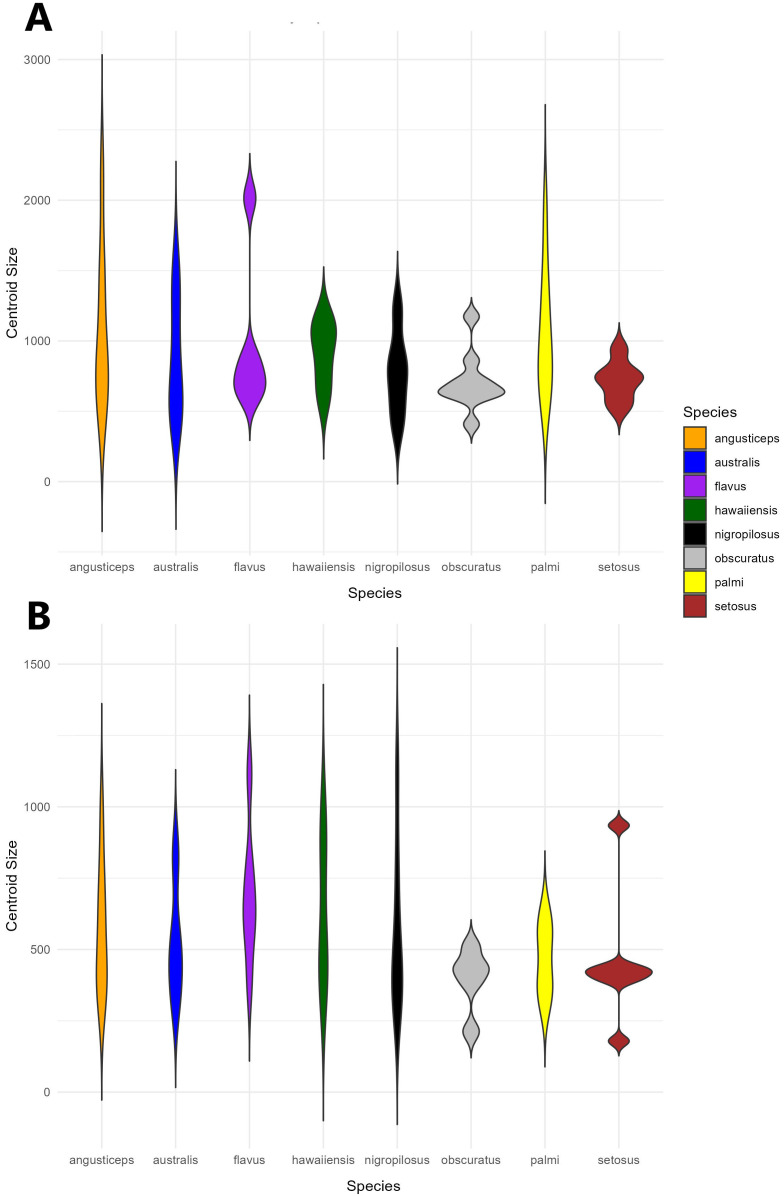
Violin plot showing the distribution of centroid size by species for **(A)** head shape and **(B)** thorax shape. The width of the violin indicates a higher density of individuals.

A violin plot of centroid size distribution showed that four species exhibited greater variance in head size, with *T. angusticeps* displaying both the largest size and the highest variance. In contrast, *T. palmi*, *T. australis*, and *T. flavus* had smaller sizes and lower variance. Notably, *T. setosus* showed minimal variance in head size (**Figure 4A**).

Shape variance was statistically significant between species, as determined by permutation tests of morphological distances (**Table 1**). The largest Procrustes and Mahalanobis distances were observed between *T. angusticeps* and *T. australis*, indicating the greatest differences in head shape. In contrast, the shortest distances, reflecting the most similar head shapes, were between *T. hawaiiensis* and *T. palmi*. When analyzing Procrustes distances, *T. australis* exhibited the most distinct head shape, significantly differing (p < 0.0001) from three other species. Similarly, *T. angusticeps* showed significant differences (p < 0.0001) with two other species.

### 3.2 Mesothoracic and metathoracic setal insertions

A PCA of the covariance matrix of thorax shape revealed that meso- and metathoracic setae insertions are distinctly associated with each *Thrips* species. The first three principal components (PCs) accounted for over 70% of the variation in thorax shape (PC1 = 39.59%; PC2 = 18.94%; PC3 = 12.22%). The PCA shows clustering of individuals primarily driven by variance along PC1, in contrast to the head shape, where variance was predominantly explained by PC2. Despite the higher percentage of variance explained, the overall shape variation in the thorax was less pronounced compared to the head, with overlapping species groups observed in the upper-right corner of the morphospace. However, certain species, such as *T. hawaiiensis* and *T. obscuratus*, showed more apparent separation. Notably, the most significant variance along PC2 was attributed to specimens of *T. nigropilosus* (**Figure 2B**).

The average shape PCA reveals a similar overall pattern among specimens, with *T. nigropilosus* exhibiting the most disparate shape. In this species, the distribution of setae forms an oval pattern, with a smaller and flattened thorax. In contrast, the shape of the cluster of specimens varies depending on the position of the setae, resulting in more elongated thoraxes in *T. angusticeps* and *T. setosus*. *T. palmi* displays an arrow-shaped elongation, while *T. obscuratus* and *T. hawaiiensis* exhibit wider, more horizontally elongated thoraxes, contributing to greater disparity in these species (**Figure 3B**). ANOVA analyses revealed no significant differences in size across the species (centroid size: F = 1.75, p = 0.1224), but significant differences in shape (Procrustes distances: F = 11.47, p < 0.0001).

A violin plot of centroid size distribution indicated that five species exhibited greater variance in thorax size, with *T. nigropilosus* displaying both the largest size and the highest variance. In contrast, *T. obscuratus* and *T. palmi* showed smaller sizes with lower variance. Similar to the head, *T. setosus* exhibited minimal variance, although its sizes were distributed into three distinct groups (**Figures 4A, B**).

Finally, shape variance was found to be statistically significant among species, as determined by permutation tests of morphological distances (**Table 2**). The largest Mahalanobis and Procrustes distances, reflecting the greatest differences in the shape formed by thoracic setal insertions, were observed between *T. angusticeps* and *T. nigropilosus*, and between *T. hawaiiensis* and *T. palmi*, respectively. In contrast, the shortest distances, indicating the most similar shapes formed by thoracic setal insertions, were found between *T. flavus* and *T. setosus*. When analyzing Procrustes distances, *T. angusticeps* exhibited the most distinct shape, as it significantly differed (p < 0.0001) from four other *Thrips* species.

**Table 2:** Morphometric Procrustes and Mahalanobis Distances of thorax shape between *Thrips* species, with respective permutation comparison p-values.

Distances	Procrustes distances						
Species	*Thrips* *angusticeps*	*Thrips* *australis*	*Thrips* *flavus*	*Thrips* *hawaiiensis*	*Thrips* *nigropilosus*	*Thrips* *obscuratus*	*Thrips* *palmi*
*Thrips australis*	0.129						
*Thrips flavus*	0.098	0.087					
*Thrips hawaiiensis*	0.220	0.196	0.157				
*Thrips nigropilosus*	0.167	0.148	0.161	0.227			
*Thrips obscuratus*	0.166	0.142	0.108	0.077	0.158		
*Thrips palmi*	0.108	0.144	0.110	0.234	0.150	0.173	
*Thrips setosus*	0.102	0.076	0.061	0.196	0.167	0.147	0.118
	Mahalanobis distances						
*Thrips australis*	8.800						
*Thrips flavus*	4.883	5.888					
*Thrips hawaiiensis*	8.49	10.333	7.087				
*Thrips nigropilosus*	11.64	7.925	10.99	13.693			
*Thrips obscuratus*	7.355	7.125	6.02	5.437	8.887		
*Thrips palmi*	6.653	7.951	6.576	10.377	9.001	7.436	
*Thrips setosus*	7.267	5.492	**4.219**	8.828	10.094	6.604	5.837

The bold number represents the non-significative value of the permutation test.

## 4 Discussion

The findings of this study clearly demonstrate the presence of significant interspecific differences within the genus *Thrips*, particularly in the morphological configurations of the head and the insertion patterns of mesothoracic and metathoracic setae. Geometric morphometrics, renowned for its precision in detecting subtle shape variations, provides a valuable tool for examining morphological differentiation among species. Unlike genetic markers, which often require more gradual genotypic changes to reveal population structure, geometric morphometrics can rapidly detect phenotypic modification, offering a practical advantage for monitoring and assessing morphological diversity. In the context of Thrips species differentiation, analyzing thorax and head shapes through geometric morphometrics enhances the resolution of species identification, especially when traditional taxonomic traits are less distinct or when closely related species exhibit convergence due to ecological or evolutionary pressures. There are very few works using the study of shape to separate among genera or species of thrips; most recently, a published work by Smith-Pardo (27) found that there were statistically significant differences among the pronotal shapes of phytophagous genera of thrips in the subfamily Phlaeothripinae; before that, there was only the work by Dos Santos et al. (28) which used geometric morphometrics to separate *Gynaikothrips uzeli* (Zimmerman) from *Gynaikothrips ficorum* (Marchal) as well as their sexes; other works involved traditional morphometry (size and proportions) in distinguishing among some species of thrips.

Although the results shown here only present a limited number of species and specimens in the same genus of thrips, they provide good evidence that geometric morphometrics can be used to discriminate among closely related species in the genus, which can sometimes be challenging to identify, in particular when such groups are highly diverse and speciose, as in the genus *Thrips* (4). Our findings highlight the utility of geometric morphometrics in disentangling complex morphological relationships between species, even when size differences are not significant. The clear shape differentiation revealed by the morphometrics distances, along the patterns captured in morphospace through PCA, point out the utility of GM for species identification. This precision is critical not only for distinguishing morphologically similar taxa but also for understanding their morphological traits’ ecological and evolutionary implications (29–31). By providing a robust framework for analyzing subtle but meaningful variations, geometric morphometrics advances species-level taxonomy and the broader study of morphological adaptations in response to ecological pressures (32). Our results highlight the utility of landmark-based analyses and the importance of employing complementary sets of landmarks to achieve robust species-level differentiation. In cases where one set of landmarks is insufficient to reveal statistical differences, another may provide the necessary resolution. For instance, *T. palmi* (a quarantine-significant species) and *T. hawaiiensis* (a common, non-quarantine-significant species in the USA) exhibit no statistically significant differences in head shape but show significant differences in the shape formed by the insertion of mesothoracic and metathoracic setae. Conversely, other species pairs demonstrate the reverse pattern, with significant differences in head shape but not in the thoracic setal insertion patterns. These findings underscore the complementary nature of multiple landmark sets in capturing species-specific morphological variations, enhancing the accuracy and reliability of geometric morphometric analyses.

As demonstrated in *Thrips* species and other taxa, landmark-based analyses provide a robust framework for distinguishing closely related species (30, 33–36). These methods enable the quantification of shape variations, as highlighted in studies of *Nyctelia* beetles, where cryptic species designations were challenged by detecting subtle morphological differences that traditional approaches had overlooked​ (30). Similarly, geometric morphometrics have revealed that complementary landmark sets, such as those used for head and thoracic morphology in Thrips, can uncover significant interspecific differences, even when individual sets alone are insufficient. This capability underscores the potential of geometric morphometrics not only in resolving taxonomic challenges but also in refining the concept of cryptic species by addressing methodological limitations. Furthermore, as recently discussed by Smith-Pardo et al. (in press), even in cases where discrete external morphological characters are commonly used in traditional taxonomy, these may not be as helpful for taxa where there is extensive convergence or constraint due to sharing highly similar ecological habits or life history strategies, such as in the case of phytophagous thrips that feed on leaves and flowers, in this case, GM can be helpful when dealing with complex taxonomic problems associated with similar, external morphological characters.

It is also important to highlight that the application of GM enhances scientific rigor in describing critical aspects of phenotypic dimension, as emphasized by Viscosi and Cardini (37). Finally, geometric morphometrics serves as a powerful tool for the identification of species within closely related groups based on external morphology. Moreover, its application in distinguishing quarantine-significant species from other morphologically similar taxa offers a fast, economical, and reliable method for pest identification. By integrating advanced analytical techniques like geometric morphometrics into traditional taxonomic frameworks, we can bridge the gap between morphology and genetics, paving the way for more precise and efficient species identification.

## Data Availability

The raw data supporting the conclusions of this article will be made available by the authors, without undue reservation.

## Author's note

The findings and conclusions in this publication have not been formally disseminated by the U.S. Department of Agriculture and should not be construed to represent any Agency determination or policy. Mention of trade names or commercial products in this publication is solely to provide specific information and does not imply recommendation or endorsement by the U.S. Department of Agriculture. USDA is an equal opportunity provider and employer.
